# Metabolomics studies in common multifactorial eye disorders: a review of biomarker discovery for age-related macular degeneration, glaucoma, diabetic retinopathy and myopia

**DOI:** 10.3389/fmolb.2024.1403844

**Published:** 2024-08-13

**Authors:** Gizachew Tilahun Belete, Lei Zhou, King-Kit Li, Pui-Kin So, Chi-Wai Do, Thomas Chuen Lam

**Affiliations:** ^1^ Centre for Myopia Research, School of Optometry, The Hong Kong Polytechnic University, Kowloon, Hong Kong SAR, China; ^2^ Research Centre for SHARP Vision (RCSV), The Hong Kong Polytechnic University, Kowloon, Hong Kong SAR, China; ^3^ Centre for Eye and Vision Research (CEVR), The Hong Kong Polytechnic University, Kowloon, Hong Kong SAR, China; ^4^ Department of Applied Biology and Chemical Technology, The Hong Kong Polytechnic University, Kowloon, Hong Kong SAR, China; ^5^ University Research Facility in Life Sciences, The Hong Kong Polytechnic University, Kowloon, Hong Kong SAR, China; ^6^ Research Centre for Chinese Medicine Innovation (RCMI), The Hong Kong Polytechnic University, Kowloon, Hong Kong SAR, China

**Keywords:** metabolomics, biomarkers, metabolic pathways, AMD, glaucoma, DR, myopia

## Abstract

**Introduction:**

Multifactorial Eye disorders are a significant public health concern and have a huge impact on quality of life. The pathophysiological mechanisms underlying these eye disorders were not completely understood since functional and low-throughput biological tests were used. By identifying biomarkers linked to eye disorders, metabolomics enables early identification, tracking of the course of the disease, and personalized treatment.

**Methods:**

The electronic databases of PubMed, Scopus, PsycINFO, and Web of Science were searched for research related to Age-Related macular degeneration (AMD), glaucoma, myopia, and diabetic retinopathy (DR). The search was conducted in August 2023. The number of cases and controls, the study’s design, the analytical methods used, and the results of the metabolomics analysis were all extracted. Using the QUADOMICS tool, the quality of the studies included was evaluated, and metabolic pathways were examined for distinct metabolic profiles. We used MetaboAnalyst 5.0 to undertake pathway analysis of differential metabolites.

**Results:**

Metabolomics studies included in this review consisted of 36 human studies (5 Age-related macular degeneration, 10 Glaucoma, 13 Diabetic retinopathy, and 8 Myopia). The most networked metabolites in AMD include glycine and adenosine monophosphate, while methionine, lysine, alanine, glyoxylic acid, and cysteine were identified in glaucoma. Furthermore, in myopia, glycerol, glutamic acid, pyruvic acid, glycine, cysteine, and oxoglutaric acid constituted significant metabolites, while glycerol, glutamic acid, lysine, citric acid, alanine, and serotonin are highly networked metabolites in cases of diabetic retinopathy. The common top metabolic pathways significantly enriched and associated with AMD, glaucoma, DR, and myopia were arginine and proline metabolism, methionine metabolism, glycine and serine metabolism, urea cycle metabolism, and purine metabolism**.**

**Conclusion:**

This review recapitulates potential metabolic biomarkers, networks and pathways in AMD, glaucoma, DR, and myopia, providing new clues to elucidate disease mechanisms and therapeutic targets. The emergence of advanced metabolomics techniques has significantly enhanced the capability of metabolic profiling and provides novel perspectives on the metabolism and underlying pathogenesis of these multifactorial eye conditions. The advancement of metabolomics is anticipated to foster a deeper comprehension of disease etiology, facilitate the identification of novel therapeutic targets, and usher in an era of personalized medicine in eye research.

## 1 Introduction

Globally, at least 2.2 billion individuals have visual impairment, of which half could have been prevented or has yet to be addressed. Ocular disorders like cataract, refractive error, age-related macular degeneration (AMD), glaucoma, and diabetic retinopathy (DR) contributed to nearly 1 billion cases of visual impairment or blindness (Steinmetz et al., 2021). The natural disease courses of common eye disorders are often complicated and interrelated. These disorders have been linked to a number of genetic and environmental factors, but little is known about how these variables interact or play a role in pathogenic pathways (Shastry, 2011). There are a wide range of anatomical, physiological, and molecular changes in different eye disorders. These changes can vary depending on the specific eye disorder and its severity. Some common anatomical changes include alterations in the morphology and structure of the eye, while physiological changes may involve impaired visual functions. Moreover, molecular changes occur at a cellular level, affecting the functioning of various proteins, metabolites, and signaling pathways within the eye (Mataruga and Müller, 2014; Hameed et al., 2020).

Blindness and visual impairment caused by various eye disorders have a profoundly severe impact on the quality of life for many adults and teenagers that have emerged as major public health issues (Ferris and Tielsch, 2004). The pathophysiological processes behind eye disease are not completely understood due to limited information on these kinds of disorders obtained via low-throughput biological testing and subsequent functional testing of candidate genes, metabolites, and proteins. A comprehensive approach to physiological and molecular alterations must thus be examined in a hypothesis-free manner, ensuring high-throughput analysis. Different omics, which is the broad characterization and measurement of biological molecules, have developed rapidly over the years because of scientific advances in mass spectrometry, sequencing, and bioinformatics (Grochowski et al., 2020; Chauhan et al., 2022).

To improve the prognosis for eye diseases, it is crucial to develop more efficient screening methods and/or diagnostic biomarkers. Thus, metabolomics is a potential method for identifying numerous biomarkers to further advance our knowledge on the etiology of eye diseases. By studying the metabolic profile of individuals with eye diseases, researchers can identify specific biomarkers that are associated with these conditions. These biomarkers could then be used for early detection, monitoring disease progression, and developing targeted therapies. Moreover, metabolomics has laid a basic foundation to understand the underlying mechanisms and impacted pathways in eye diseases, paving the way for more effective treatments in the future.

Different studies have examined the relationship between common ocular disorders and metabolite changes in the eye. It raises questions about the clear map of potential connections between metabolites and ocular disorders since metabolites may have different expression profiles in different ocular conditions. This review provides a better and more comprehensive understanding of how alterations in metabolites can affect ocular health in disease conditions.

## 2 Overview of metabolomics

Metabolites are low molecular weight organic compounds that participate in chemical processes within the cells. They are required for numerous biological functions, including energy generation, signaling, and gene expression control (Mamas et al., 2011). In 1998, the term metabolome was first used in relation to the genome, transcriptome, and proteome. Shortly after, the first academic articles employing the phrases metabolomics or metabolic profiling were released (Alseekh and Fernie, 2018). The study of metabolites in a biological system and how they vary in response to diverse situations is known as metabolomics. The fundamental goal of the metabolomics sciences is to discover, characterize, and quantify the biomolecules and molecular processes that affect the structures and functions of cells and tissues. Metabolomics has grown in importance as a technique for studying the underlying causes of numerous diseases, such as cancer, diabetes, and degenerative and neurological disorders. Researchers can gain insight into the metabolic pathways that are linked to these disorders by studying the metabolites present in various biological samples such as blood, urine, and tissue (Idle and Gonzalez, 2007).

Metabolomics, while still in its early stages compared to genomics and proteomics, is quickly becoming an essential tool in medicine. This study of the metabolome is particularly relevant to the phenotype, providing insights into normal and diseased states, as well as responses to external stimuli. It serves as a dynamic indicator of genetic, environmental, or disease-related disruptions and offers a sensitive measure of disease phenotype due to the direct association of metabolites with biological processes (Dunn et al., 2011; Trivedi et al., 2017). Metabolomics offers numerous advantages in the medical domain, such as identifying biomarkers for disease risk prognosis and diagnosis, evaluating disease progression, elucidating the impact of environmental and lifestyle factors on disease, and assessing drug efficacy, toxicity, and adverse reactions. Furthermore, metabolomics exhibits stronger associations with disease outcomes than genetics and requires fewer samples for investigation compared to genetic research (Nicholson et al., 2012; Jové et al., 2014).

**FIGURE 1:**
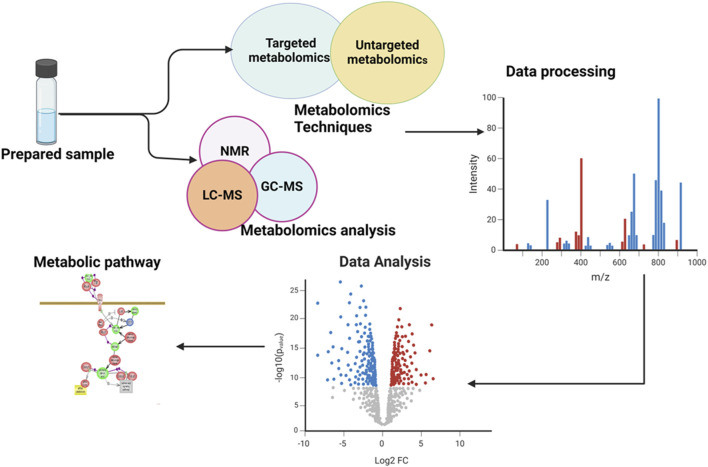
Illustration of an overview of the workflow involved in a metabolomics study. The figure showed the comprehensive processes and provided a holistic overview of the experimental workflow and analytical procedures employed.

Ocular metabolomics has the potential to revolutionize the identification and treatment of eye disorders by uncovering metabolic processes in ocular tissues and fluids. This knowledge can lead to the development of early diagnosis and interventions, as well as the identification of specific metabolites associated with various eye conditions, which used as biomarkers in diagnostic assays (Young and Wallace, 2009; Tan et al., 2016). It also aids in tracking the development of diseases and evaluating the efficacy of therapies by examining changes in metabolite levels over time. Additionally, ocular metabolomics may reveal individual variations in metabolite profiles, which may be exploited to create patient-specific therapy regimens (Bobadilla et al., 2022). The general overview of metabolomics is illustrated in the following Figure 1.

## 3 Development of metabolomics and its technological advancement

In the late 1940s, Roger Williams put forward an idea that suggested each person has a unique metabolic profile represented in their bodily fluids. With a curiosity to identify metabolic pattern variances across different individuals with schizophrenia, he conducted a paper chromatography test by examining metabolic components found in bodily fluids such as saliva and urine (Williams and Berry, 1951). Technological advancements have made it possible to quantitatively measure metabolites. In 1971, Horning and colleagues demonstrated the feasibility of quantifying chemicals in tissue extracts and urine using gas chromatography-mass spectrometry (GC-MS) and coined the term “metabolic profile” (Horning and Horning, 1971). Concurrently, nuclear magnetic resonance (NMR) technology began detecting metabolites in raw biological samples. Advancements in magnetic field strength and magic angle spinning subsequently heightened the technology’s sensitivity. Nicholson et al. demonstrated the potential for using NMR spectroscopy to diagnose diabetes mellitus in 1984 (Nicholson et al., 1983).

Growing evidence highlights the significance of metabolism in various diseases and medical conditions. Metabolomics offers unprecedented value to fundamental drug research. This value encompasses the identification of drug targets, understanding disease etiology and mechanisms, and notably expedites drug development by predicting pharmacokinetics, pharmacodynamics, and therapeutic response. Additionally, metabolomics facilitates the exploration of medication interactions, drug repurposing, and the development of personalized treatment strategies (Pang and Hu, 2023). Following significant progress, the primary methods for capturing and analyzing metabolomes in metabolomics profiling are NMR and mass spectrometry (MS). Effective sample preparation and data analysis have also played a crucial role in advancing metabolomics alongside instrumentation.

### 3.1 Nuclear magnetic resonance spectroscopy

Nuclear magnetic resonance is a non-destructive technique that provides information about the chemical structure and dynamics of metabolites. Based on their chemical shifts and coupling constants, metabolites may be identified and quantified using NMR (Emwas et al., 2019). The foundation of NMR is the idea that specific atomic nuclei’s magnetic properties can be applied to infer information about the physical and chemical characteristics of the atoms or molecules they are included in. In a magnetic field, magnetic nuclei absorb and re-emit electromagnetic radiation at a specific resonance frequency determined by the intensity of the magnetic field and the atom’s isotope’s magnetic characteristics (Dunn et al., 2011).

The analysis of intact tissue samples in solid form is also possible using high-resolution magnetic angle spinning (MAS) NMR. The ordinarily wide spectrum could be made smaller by rotating a sample quickly at the magic angle (m = 54.74°) with regard to the magnetic field’s direction (Midelfart, 2009; Moestue et al., 2011). Kryczka et al. conducted the first MAS NMR analysis of human eye tissues and identified 29 metabolites. The study revealed a correlation between tissue biochemical composition and biological activities (Alia et al., 2009).

NMR-based experiments for fluid samples like urine, serum, plasma, and CSF require less preparation compared to MS investigations. While MS provides molecular formula information, NMR can distinguish structural isomers. Despite being less sensitive and requiring larger sample quantities, NMR spectroscopy offers high quantity, repeatability, and non-destructiveness to tissue samples, allowing for multiple tests on the same sample (Dunn et al., 2011). NMR spectroscopy has the benefit of avoiding sample derivatization, which may introduce artifacts and decrease the precision of metabolite measurement. NMR spectroscopy may still be used to identify low-abundance metabolites, although it does so to a lesser extent than MS due to its reduced sensitivity (Johnson and Gonzalez, 2012). By combining these two techniques, researchers can obtain comprehensive insights into the metabolome and gain a deeper understanding of metabolic pathways and disease mechanisms (Ashrafian et al., 2021).

### 3.2 Mass spectrometry

The mass of a molecule is determined using mass spectrometry (MS) by analyzing the mass-to-charge ratio (m/z) of its ions. Mass spectrometers operate in positive and negative ion modes to identify ionized species. Peaks in the resulting mass spectra can provide information about the molecular weight and, in some cases, the structural composition of the sample (Dunn et al., 2011).

Gas chromatography-mass spectrometry (GC-MS), liquid chromatography-mass spectrometry (LC-MS), capillary electrophoresis mass spectrometry (CE-MS) and Ion chromatography-mass spectrometry (IC-MS) are common pre-mass spectrometer separation techniques that enhance sensitivity and facilitating metabolite identification (Lu et al., 2008). GC separates based on volatility or other chemical characteristics, while LC relies on polarity, with hydrophilic interaction liquid chromatography (HILIC) and reversed-phase (RP) methods being common. Capillary electrophoresis separates based on polarizability and molecular shape (Muller and de Villiers, 2023). Likewise, Ion chromatography (IC) is an analytical method that separates analytes through ionic exchange, commonly used for both anion and cation analysis. IC is effective in detecting small inorganic ions such as phosphate, nitrate, and sulfate ions, as well as in analyzing polar metabolites like uric acid and amino acids (Amin et al., 2008).

Initially, mass spectrometry was used to identify and quantify individual metabolites in a sample. However, with the development of more advanced techniques, mass spectrometry can now be used to analyze entire metabolic pathways and networks (Dunn, 2008). One major advance in MS for metabolomics is the development of high-resolution mass spectrometry (HRMS). HRMS’s ability to deliver high mass accuracy allows for more reliable identification of unknown compounds (Nazifova-Tasinova et al., 2020).Overall, the use of MS in metabolomics has become an essential tool for studying metabolic pathways and networks and has greatly advanced our understanding of metabolism in health and disease (Chen et al., 2018; Alarcon-Barrera et al., 2022). Metabolomics in eye research facilitates the identification of disease-related biomarkers which can be used as diagnostic, screening, or prognostic tools in the clinical setting.

### 3.3 Sample preparation for metabolomics

The package of metabolomics advancements has revolutionized sample preparation to provide plausible outcomes, leading to more reliable and meaningful results The choice of sample preparation methods is dependent on the purposes of the investigation. Commonly employed methods include liquid-liquid extraction (LLE), solid-phase extraction (SPE), and protein precipitation (PP) (Kole et al., 2011). In LLE, the sample is mixed with a solvent to draw out the desired metabolites, and the mixture is subsequently centrifuged to separate the solvent from the sample matrix. SPE entails eluding the metabolites from a solid-phase cartridge before washing it to get rid of additional matrix components. More importantly, for the analysis of metabolites with particularly low abundance, high susceptibility to matrix interference, and particularly high or low polarity, LLE and SPE are applied for the purposes of sample enrichment and matrix removal. PP is the simplest sample preparation method and mainly applied for general profiling. It separates proteins from biological materials by adding a precipitating agent to the sample and centrifuging to separate protein precipitate from the sample matrix (Li et al., 2019).

### 3.4 Data analysis

An essential and integral part of metabolomics research is data analysis. High-throughput NMR and MS spectrum data must undergo preprocessing to ensure their quality and reliability. During metabolomics data analysis, there are various steps including peak picking, retention time alignment, intensity normalization, compound identification and statistical analysis (Ren et al., 2015). In metabolomics research, the tasks of peak picking and retention time alignment are commonly addressed using software tools such as XCMS, MZmine, and OpenMS. Similarly, intensity normalization procedures are typically conducted utilizing tools like MetaboAnalyst, MetaboLights, and MZmine. Furthermore, the process of compound identification is facilitated by software solutions such as MetFrag, mzCloud, and MassBank, among others (Misra and van der Hooft, 2016). Subsequent to the steps involving filtering, recognition, identification, quantification, scaling, and normalization of metabolite properties, data analysis is often carried out either on the spectra themselves or on the resulting concentration table. Lastly, the statistical analysis of metabolomics data is commonly executed using software platforms like MetaboAnalyst, MetaboLights, SIMCA, or other tools (Chang et al., 2021).

Both parametric and nonparametric tests, including the *t*-test, analysis of variance (ANOVA), Mann Whitney U, and Kruskal Wallis one-way analysis, can be employed to identify biomarkers associated with the desired outcome (Vinaixa et al., 2012). Unsupervised multivariate statistical methods such as principal component analysis (PCA), self-organizing maps (SOM) and hierarchical cluster analysis (HCA) are valuable for exploring patterns and clusters, as well as for addressing data quality concerns such as outliers and batch effects. Concentration tables and spectra can serve as inputs for supervised algorithms like partial least square discriminant analysis (PL-SDA) and orthogonal PLS-DA to make predictions, identify biomarkers, and distinguish between disease phenotypes and endotypes (Worley et al., 2013). For large datasets, machine learning techniques such as hidden Markov models, Bayesian methods, support vector machines (SVM), random forests, and neural networks can be beneficial for prediction and biomarker discovery (Liebal et al., 2020).

## 4 Metabolomics approaches

The discipline of health and biomedical sciences has one of the most interesting applications for metabolomics. An individual’s specific metabolic profile may be determined by examining the metabolites found in their biological samples. In a single examination, hundreds to thousands of metabolites may be assessed, but their identities might not be known before or after the investigation (Hotea et al., 2023). Metabolomics data’s volume and complexity often require high-performance bioinformatics tools for post-processing and analysis. Metabolomics analysis can encompass a wide range of samples, including tissue, tears, blood, urine, sweat, and cultured cells or media (Gonzalez-Covarrubias et al., 2022).

Metabolomics employs two approaches: targeted and untargeted, both capable of detecting biomarkers but facing constraints in clinical research. Proper sample preparation, including extraction, purification, and derivatization, is essential to remove interfering substances and enhance metabolite detection, depending on the type, quantity, and intended strategy of the samples (Lindon et al., 2011).

Untargeted metabolomics is a comprehensive examination of a biological entity’s metabolite composition under certain physiological circumstances. Untargeted metabolomics is usually used to develop hypotheses related to metabolic changes (Schrimpe-Rutledge et al., 2016). However, it is hard to cover all metabolites objectively owing to the limits of existing analytical platforms and the requirements for sample collection and processing (Fiehn, 2002).

Untargeted metabolomics research can uncover new metabolic processes because of their global character; however, processing enormous volumes of raw data, challenges in recognizing and characterizing unknown small molecules, dependence on the platform’s inherent analytical coverage, and bias towards the detection of high-abundance compounds are the key drawbacks of untargeted metabolomics (Roberts et al., 2012; Dunn et al., 2013).

Targeted metabolomics measures specific groups of metabolites related to chemical composition or biological function, aiding in the quantification of known compounds, such as in drug metabolism studies. This approach leverages metabolic kinetics, end products, routes, and biochemical pathways to generate hypotheses and gain deeper insights from untargeted investigations (Ioannidis and Khoury, 2011). Low-abundance compounds are favored by techniques like the triple quadrupole mass spectrometer (TQMS), which also enables the measurement of metabolites with low concentrations. It is also possible to minimize high-abundance compounds during sample preparation. With more selectivity and sensitivity than untargeted techniques, targeted metabolomics examines a limited number of metabolites (Patti et al., 2012).

## 5 Methods

### 5.1 Search strategy

**FIGURE 2:**
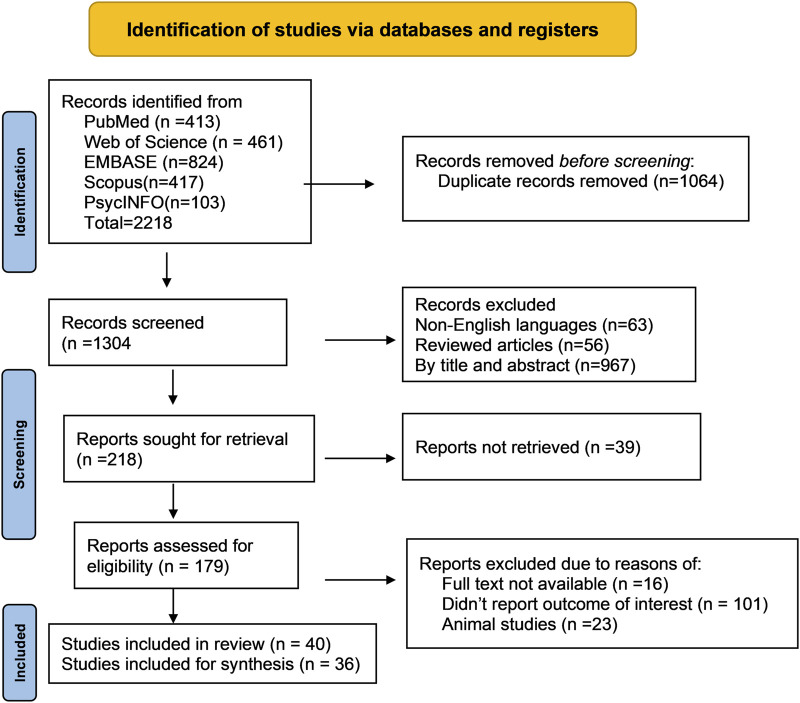
Flow diagram of literature search and study selection for metabolomics of common eye disorders. This portrays the exploration of libraries, the procedures for literature review, the selection criteria, and the selection process.

We conducted a systematic review of metabolomics studies pertaining to common ocular disorders (myopia, age-related macular degeneration, glaucoma, and diabetic retinopathy) based on the recommendations of the Meta-analysis of Observational Research in Epidemiology (DS, 2000). The reporting of the study’s findings adhered to the Preferred Reporting Items for Systematic Review and Meta-Analysis (PRISMA) standards (Liberati et al., 2009) (Figure 2). We used the following search terms: ((“metabolomics” OR “metabonomics” OR “metabolome” OR “metabolites” OR “metabolic profiling”)) AND ((“myopia” OR “short sightedness” OR “myopic degeneration”) OR (“macular degeneration” OR “age-related maculopathy” OR “age-related macular degeneration”) OR (“glaucoma” OR “ocular hypertension” OR “glaucomatous optic neuropathy”) OR (“diabetic retinopathy” OR “diabetic eye disease” OR “diabetic macular edema” OR “diabetic eye complications”) OR (“ocular disorders” OR “eye diseases”)) to discover relevant metabolomics studies published up to August 2023. In specific to EMBASE, the search term was modified as (“metabolomics”/exp OR “metabolomics” OR “metabonomics”/exp OR “metabonomics” OR “metabolome”/exp OR “metabolome” OR “metabolites”/exp OR “metabolites“ OR “metabolic profiling”/exp OR “metabolic profiling”) AND (“myopia”/exp OR “myopia” OR “short sightedness”/exp OR “short sightedness” OR “myopic degeneration” OR “macular degeneration”/exp OR “macular degeneration” OR “age-related maculopathy” OR “age-related macular degeneration”/exp OR “age-related macular degeneration” OR “glaucoma”/exp OR “glaucoma” OR “ocular hypertension”/exp OR “ocular hypertension” OR “glaucomatous optic neuropathy”/exp OR “glaucomatous optic neuropathy” OR “diabetic retinopathy”/exp OR “diabetic retinopathy” OR “diabetic eye disease”/exp OR “diabetic eye disease” OR “diabetic macular edema”/exp OR “diabetic macular edema” OR “diabetic eye complications”/exp OR “diabetic eye complications” OR “ocular disorders” OR “eye diseases”/exp OR “eye diseases”). The discovered articles were imported into citation management software (EndNote version 20, Clarivate, London, United Kingdom) for further screening and evaluation. Two authors conducted separate searches for publications and filtered those that were included based on the title and abstract.

### 5.2 Inclusion and exclusion criteria

We carried out a thorough analysis of all research written in English to determine if they used a human metabolomics technique based on nuclear magnetic resonance (NMR) or mass spectrometry (MS). Studies were excluded if they used animals or if they didn't provide adequate metabolic data.

### 5.3 Quality assessment

QUADOMIC’s technique was used to evaluate the quality of the studies (Lumbreras et al., 2008). It is an adaptation of the QUADAS tool, which considers the challenges posed by omics and is used to systematically evaluate diagnostic accuracy investigations. QUADOMICS combines four programs that address sample characteristics, preanalytical, clinical, and physiological differences in study participants, as well as overfitting in data collection and analysis.

The data were collected and synthesized for each paper, encompassing the quantity of biological samples, detection and analysis tools, sample size, study design and procedures, and repeated biomarker reports. Employing MetaboAnalyst software (version 5.0) (Pang et al., 2021), pathway analysis and topological findings were elucidated based on the anticipated disease-influencing metabolites reported in all the papers included in the review. The pathway impact value was obtained from the examination of the pathway topology, while the calculated *P*-value was based on the analysis of the pathway enrichment.

## 6 Application of metabolomics in eye diseases

In human eyes, ocular fluids and tissues are rich in metabolites. The common sources of metabolomics samples are retina, aqueous humor, vitreous, and tear film. Drawings of the aqueous and vitreous humor are intrusive procedures, and patients having eye surgery are eligible to provide these samples. Studying ocular conditions where the blood-retinal-barrier is compromised, such as DR or AMD, could be helpful with plasma or serum metabolomics studies (Luo et al., 2020). There are common or distinct ranges of anatomical, physiological, and metabolic changes in the retina during the course of AMD, glaucoma, myopia, and DR. These changes give clues to consider the retina as a primary target for many researchers to investigate disease biomarkers. The retina is rich in interphotoreceptor matrix, which is a proteoglycan-based substance used to exchange metabolites between the retinal pigment epithelium (RPE) and other layers (Hurley et al., 2015).

Due to the blood-aqueous and blood-retinal barriers, the eye has a unique metabolome, which makes it a perfect organ for metabolomics studies. Aqueous and vitreous humor’s metabolomes could reflect local metabolism, with the systemic environment having only a modest impact (Zhang et al., 2023). Characterizing healthy biofluids, researching tissue metabolism, comprehending disease causes, and developing novel treatments for eye diseases have all been realized in eye care by using metabolomics. Finding disease-related biomarkers or risk variables, which can be used as diagnostic, screening, or prognostic tools in the clinical setting, is an important clinical application. More studies are still needed to define the normal metabolome and establish what constitutes normal intra- and inter-individual variability. The main focus of this review is the application of metabolomics in AMD, glaucoma, DR and myopia.

### 6.1 Age-related macular degeneration (AMD)

By far, age-related macular degeneration (AMD) is a very common eye disease causing permanent visual impairment over the age of 65 years, accounting for 8.7% of blindness across the globe (Friedman BJOC et al., 2004). Currently, there are an estimated 196 million individuals affected by AMD, with projections indicating a rise to 288 million by 2040 (Wong et al., 2014). There are two main types of AMD: dry AMD and wet AMD. Dry AMD represents the predominant manifestation of AMD, encompassing approximately 85%–90% of all instances (Flaxel et al., 2020). Early AMD stages involve intracellular lipofuscin accumulation (drusen) in the RPE and the formation of sub-RPE deposits within the macula. Progressive deposition of drusen over time may result in macular atrophy and desiccation, culminating in a gradual decline in visual acuity (Spraul et al., 1999). Wet AMD occurs when abnormal blood vessels grow underneath the retina and leak blood and fluid into the macula, causing rapid and severe vision loss. Choroidal neovascularization (CNV), the most severe form of advanced wet AMD, leads to rapid central vision loss, while geographic atrophy (GA), another severe type, is characterized by gradual central vision decline due to RPE and photoreceptor cell degeneration (Flaxel et al., 2020). Vascular endothelial growth factor (VEGF) antibodies may attenuate CNV progression by retarding the growth of new vessels, their effects are often temporary, with the condition frequently progressing to macular atrophy following anti-VEGF therapy (Chakravarthy et al., 2013). Clinical trials suggested that the potential contribution of complement system dysregulation to AMD has led to the emergence of complement inhibition as a therapeutic strategy for slowing the progression of GA (Desai and Dugel, 2022; Spaide and Vavvas, 2023).

AMD-related alterations in lipid metabolism and fatty acid composition have been found using metabolomics studies (Suzumura et al., 2020; Heckel et al., 2022). Lipid metabolomics has shown that the amounts of different lipids, such as phospholipids, sphingolipids, and triglycerides, are altered in the blood and retina of people with AMD. Omega-3 fatty acid downregulation has been linked to the onset and development of AMD. It has been shown by the significant lipid buildup that causes drusen to develop in the macula of those with AMD. Furthermore, AMD has been linked to elevated cholesterol and atypical lipoprotein metabolism (Landowski and Bowes, 2022).

In two separate studies using serum and plasma samples, metabolite analysis of AMD patients found significant differences between cases and controls. The levels of several metabolites, such as amino acids, lipids, and carbohydrates, changed as a result of these alterations. Differential glycerophospholipid metabolism, lipid super-pathway, and amino acid metabolism (including N-acetylasparagine, a component of alanine and aspartate) were found in a comparative investigation of AMD patients (Lains et al., 2018). The changed metabolite profiles point to possible disturbances in the retina’s metabolic pathways, which could help AMD advance and progress (Lains et al., 2018; Shen et al., 2022).

In a targeted metabolomics analysis of samples from the advanced AMD group showed upregulation of bi- and tripeptides, covalently modified amino acids, bile acids, vitamin D-related metabolites, lipoproteins, and their subclasses (cholesterols, glycerides, and phospholipids) (Osborn et al., 2013). These also suggested dysregulation in various metabolic pathways in the advanced AMD group. The presence of covalently modified amino acids and bile acids may indicate oxidative stress and inflammation in AMD patients (Osborn et al., 2013; Sim et al., 2022).

In a serum sample of neovascular AMD, carnitine shuttle pathways were significantly increased. This upregulation of carnitine shuttle pathways plays a crucial role in the pathogenesis of neovascular AMD (Mitchell et al., 2018). Downregulation of amino acids (Alanine, Isoleucine, Leucine, Phenylalanine, and Tyrosine) and citrate levels were reported in AMD patients compared to their counter-controls (Acar et al., 2020). Depleted citrate and amino acids showed levels in AMD, which reflects an enhancement in energy requirements in the disease process (Lains et al., 2018; Acar et al., 2020). Table1 provides a summary of metabolomics studies and significantly changed metabolites observed in AMD from serum and plasma samples.

**TABLE 1:** Summary of metabolomics studies on AMD, investigating metabolites in human biofluids, methodologies and analytical techniques.

Diseases/Condition	Sample/sample sources	Case (treatment) group	Control group	Type/study duration	Analytical technique	Metabolomics techniques	Number of identified metabolite	Key findings	Evaluation standards	References
All stages (AMD)	Plasma	n = 90 (30 with early AMD, 30 with intermediate AMD, and 30 with late AMD)	n = 30	1.42 years	UPLC-tandem MS	Untargeted	87 significant metabolites were identified	After controlling confounders, 87 metabolites were associated with AMD. Among them 72 belonged to the lipid superpathway, followed by amino acids (5) including N acetylasparagine, a component of alanine and aspartate metabolism) 59 metabolites decreased & 28 metabolites elevated	PCA, ROC analysis	Lains et al. (2018)
AMD	serum and plasma	n = 423(no control)	n = 6947	7 years	NMR	not reported	228 blood metabolites were quantified	32 blood lipid-related metabolites identified (lipoproteins and their subclasses, cholesterols, glycerides, and phospholipids) 4 single nucleotide polymorphisms	OR	Sim et al. (2022)
Neovascular AMD	Serum	n = 100 NVAMD patients	n = 192 controls	9 years	HR LCMS C18 column	Untargeted	159 metabolites distinguished NVAMD patients	110 metabolites were increased and 49 were decreased in the plasma of NVAMD patients compared to controls. Carnitine shuttle pathway were significantly increased	PLS-DA	Mitchell et al. (2018)
Neovascular AMD& polypoidal choroidal vasculopathy	Serum	n = 219 patients	no control	2 years	UHPLC-MS/MS Waters BEH C8 column	Untargeted	248 metabolites were detected	85 differential metabolites were identified, including sub-classes of diacylglycerophosphocholines, lysophosphatidylcholine (LPC), fatty acids, phosphocholine, etc. that can discriminate NAMD	PCA, OPLS-DA, FDR, FC, M-W *U* test, ROC, AUR	Shen et al. (2022)
Neovascular AMD	Plasma	n = 26 NVAMD patients	n = 19 controls	cross-sectional	LC-FTMS	Targeted	94 unique features were significant	among the 94 unique features, 40 metabolites were distinctive for NVAMD (bi- and tripeptides, covalently modified amino acids, bile acids, and vitamin D-related metabolites)	PCA, OPLS-DA,CA, FDR, SVM	Osborn et al. (2013)

#### 6.1.1 Pathway analysis for AMD-related metabolites

Metabolic pathways related to the development of AMD were discovered by an analysis using MetaboAnalyst. From this analysis, 15 metabolic pathways were considerably enriched (*p* < 0.05). All differential metabolites/biomarkers identified were used as an input set for pathway analysis.

**FIGURE 3:**
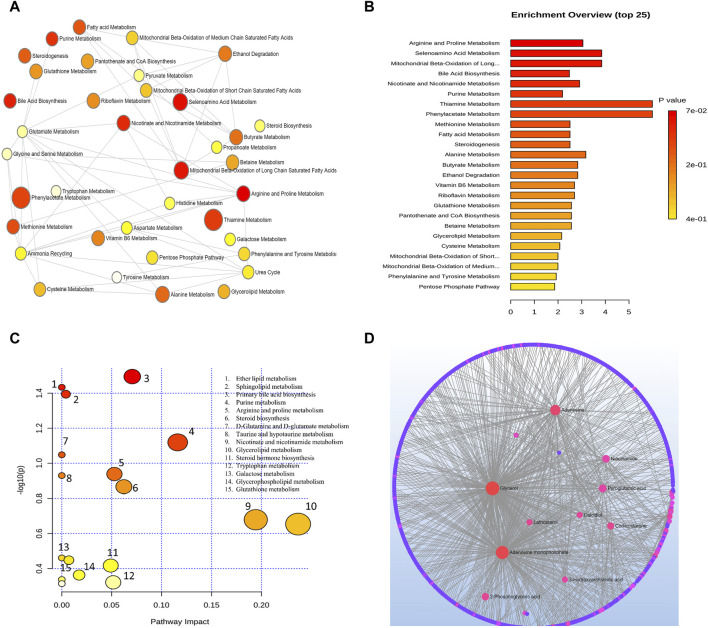
**(A)** Network view of metabolic pathway enrichment for AMD, **(B)** Summary plot of over-representation analysis (ORA) of metabolic pathways associated with AMD. The *p* values for the metabolic pathways are color coded with dark red being highly significant and white being least significant. **(C)** Overview of pathway impact analysis for AMD. The color of the circle indicates the significance level in the enrichment analysis, while the size of the circle reflects the pathway impact value from the topology analysis. **(D)** Metabolic-metabolic interaction network of AMD. The metabolite-metabolite interaction network allows for the exploration and visualization of interactions among functionally related metabolites.

Arginine and proline metabolism, selenoamino acids metabolism, long-chain saturated fatty acid mitochondrial beta-oxidation, bile acid biosynthesis, nicotinate and nicotinamide metabolism, purine metabolism, thiamine metabolism, phenylacetate metabolism, methionine metabolism, fatty acid metabolism, steroidogenesis, alanine metabolism, butyrate metabolism, and ethanol degradation metabolism were the most significant pathways as depicted in Figure 3B.

A network of metabolic pathway enrichment analyses revealed that arginine and proline, long-chain saturated fatty acid mitochondrial beta-oxidation, fatty acid metabolism, and nicotinate and nicotinamide metabolism were among the top networked metabolism pathways (Figures 3A,B). The analysis of metabolite-metabolite interaction also indicates that adenosine monophosphate, glycerol, niacinamide, adenosine, and pyroglutamic acid were exhibited in multiple networks (Figure 3D). The pathway impact of significant metabolites is represented in Figure 3C.

### 6.2 Glaucoma

Glaucoma is responsible for roughly 12% of global blindness, making it a substantial cause of permanent vision loss (Güngör et al., 2014). It is projected to impact about 111.8 million people across the globe by 2040 (Tham et al., 2014). An individual’s age, high intraocular pressure, inflammation, high myopia, and a family history of glaucoma have all been identified as factors that can increase the risk of developing this condition (Evangelho et al., 2019). Finding early detection tools is important in reducing the risk of severe visual impairment in glaucoma patients. Symptoms are not always present in the early stages of the disease and are usually present in its later stages. Researchers continue to explore new treatment targets or biomarkers to address this issue (Weinreb et al., 2014).

Preliminary research in the ophthalmology field has shown that utilizing metabolomics can reveal distinctive metabolic signatures and potential biomarkers for glaucoma. Specifically, altered metabolism of vitamins, oxidation of fatty acids, and glutaminolysis may be evident in individuals with the disease (Doganay et al., 2012; Hysi et al., 2019). For developing target-based drugs and susceptibility biomarkers, examining glaucoma metabolic profiles is critical. This exploration of differential metabolites and metabolic pathways could further aid in comprehending the pathophysiology of the disease. Such biomarkers have vast potential in diagnostic applications for this sight-threatening eye condition, although their values haven't been fully confirmed.

The synthesis of metabolites in metabolomics glaucoma studies using GC-TOF/MS and LC-TQ-MS from aqueous humor revealed that glutamate, 3-hydroxykynurenine, lactate, biotin, glucose-1-phosphate, methylmalonic acid, N-cyclohexyl formamide, sorbitol, spermidine, D-erythronolactone, dehydroascorbic acid, galactose, mannose, pelargonic acid, and ribitol were significantly altered (Chen et al., 2019; Pan et al., 2020; Pulukool et al., 2021). A non-targeted metabolomics analysis of POAG patients investigated the differential signature of plasma compared to controls. In a study employing LC-MS, metabolites such as nicotinamide, hypoxanthine, xanthine, and 1-methyl-6,7-dihydroxy-1,2,3,4-tetrahydroisoquinoline were identified as downregulated, whereas N-acetyl-L-leucine, arginine, RAC-glycerol 1-myristate, 1-oleoyl-rac-glycerol, and cystathionine were determined to be upregulated metabolites (Nzoughet et al., 2020).

Studies from plasma and serum samples unveiled potential biomarkers including cyclic AMP, 2-methylbenzoic acid, 3′-sialyllactose, hypoxanthine, uric acid, phenyl lactate, hydroxyphenyl lactic acid, barbituric acid, L-3phenyllactic acid, palmitoyl carnitine, hydroxyergocalciferol, sphingolipids, vitamin D-related metabolites, pentadecanone, heptadecanone, heptadecanediol, ergostanol, heptadecylbenzenediol, monoglyceride, and ergosterol. These metabolites underwent significant changes during the disease course, suggesting their potential as biomarkers for diagnosing and monitoring disease progression (Burgess et al., 2015; Tang et al., 2021; Kang et al., 2022; Zeleznik et al., 2023).

Targeted plasma metabolomics using LC-FIA-MS/MS on 36 POAG and 27 age-matched controls uncovered the downregulation of paspermidine, spermine, octadecadienyl-carnitine, octadecenoyl-carnitine and histamine. But other thirteen significant metabolites (tyrosine, methionine, hexoses group, phosphatidylcholine acyl-alkyl, methionine sulfoxide, propionyl-carnitine, phosphatidylcholines, butyrylcarnitine, decenoyl-carnitine, dodecenoylcarnitine and arginine) were upregulated (Leruez et al., 2018). Table 2 summarizes metabolites underwent significant change, study characteristics, and analytical techniques of glaucoma studies of human subjects.

**TABLE 2:** Summary of metabolomics studies on glaucoma, investigating metabolites in human biofluids, methodologies and analytical techniques.

Diseases/Condition	Sample/sample sources	Case (treatment) group	Control group	Type/study duration	Analytical technique	Metabolomics technique	Number of identified metabolite	Key findings	Evaluation standards	References
Primary congenital glaucoma	Aqueous humor	n = 45 PCG	n = 30 (10 congenital cataract (CC) 10 Aged-related cataract (ARC) & 10 POAG)	cross-sectional	GC/TOF MS	Untargeted	363 metabolites were identified (173 amino acid/carbohydrate/lipid/nucleotide super pathway, 132 other metabolites and 58 unknown metabolites)	89 metabolites were determined to be significantly different in PCG responsible for the separation from CC, 86 metabolites were found to be significantly changed compared to ARC group, 38 metabolites were identified to be significantly different in PCG against the control. 38 significantly different metabolites revealed enriched (aminoacyl-tRNA biosynthesis; valine, leucine, and isoleucine biosynthesis; arginine and proline metabolism; and valine, leucine, and isoleucine degradation)	PLS-DA, FDR	Chen et al. (2019)
primary open angle glaucoma	Aqueous humor	n = 6 with POAG patients	n = 11 (6 cataract patients & 5 healthy controls	cross-sectional	MRM Agilent 6490 triple quadrupole MS	Targeted	164 metabolites were determined across clinical samples of which 111 metabolites were significant	21 metabolites which are significantly different in POAG (amino acids, nucleotides, Dimethylarginine, glutamate, 3-Hydroxykynurenine, lactate, etc.) 22 metabolic pathways of multiple amino acids metabolism (glutamine, glutamate, arginine, histidine, tryptophan metabolism, etc.), purine and pyrimidine metabolism, Biotin and butanoate metabolism, sphingolipid metabolism, pyruvate metabolism as well as nicotinate and nicotinamide metabolism were identified	PCA, PLS-DA, Mann–Whitney Test	Pulukool et al. (2021)
Primary open-angle glaucoma	Aqueous humor	n = 16 patients with POAG	n = 24 cataract patients	cross-sectional	GC-TOF-MS	Untargeted	14 metabolic biomarkers were identified as potential aqueous humor biomarkers for POAG compared to controls	6 metabolites were found to be decreased in POAG (Biotin, Glucose-1-phosphate, Methylmalonic acid, N-cyclohexylformamide 1, Sorbitol, and Spermidine) but 8 metabolites (2-mercaptoethanesulfonic acid 2, D-erythronolactone 2, DTalose 1, Dehydroascorbic Acid 2, Galactose 1, Mannose, Pelargonic acid and Ribitol) increased in POAG. 5 pathways were significantly enriched (Biotin metabolism; Beta-Alanine metabolism; Glutathione metabolism; Folate biosynthesis; and Arginine and Proline metabolism)	PCA, OPLS-DA, *t*-test, AUC	Pan et al. (2020)
Primary open-angle glaucoma	Plasma	n = 34 POAG patients	n = 30 age and sex matched controls	cross-sectional	LC-HRMS	Untargeted	160 metabolites were identified for statistical analyses (28 metabolites were highlighted)	9 metabolites, namely,: nicotinamide, hypoxanthine, xanthine, and 1-methyl-6,7-dihydroxy-1,2,3,4-tetrahydroisoquinoline with decreased concentrations and N-acetyl-L-Leucine, arginine, RAC-glycerol 1-myristate, 1-oleoyl-RAC-glycerol, cystathionine with increased concentrations in POAG	CV-ANOVA, PCA, OPLS-DA, FC,AUC, SVM	Nzoughet et al. (2020)
Primary open-angle glaucoma	Plasma and Aqueous humor	n = 28 POAG patients	n = 25 controls	cross-sectional	LC-ESI MS/MS Waters Acquity UPLC HSS T3 C18	Targeted	33 significant metabolites were identified both in plasma and aqueous	22 DEM in the AH and 11 DEMs in the plasma significantly separated POAG. Cyclic AMP, 2methylbenzoic acid, 3′-sialyllactose in the AH and N-lac-phe in the plasma were identified as potential biomarkers for POAG 5 DEMs (cAMP, 2-methylbenzoic acid, hypoxanthine; xanthosine and hexadecanamide) were decreased but 17 DEMs-A (3′-sialyllactose; lysopc, dulcitol lysopc, uric acid, phenyl lactate, Hydroxyphenyl lactic acid, barbituric acid; L-3phenyllactic acid, PAF C-16, N6-succinyl adenosine, D-sorbitol etc.) were increased in POAG group	PCA, PLS-DA, ROC, AUC	Tang et al. (2021)
Primary open-angle glaucoma	Plasma	n = 72 POAG patients	n = 72 controls	cross-sectional	LC-MS	Untargeted	Of the total 2440 features recovered, 41 metabolites differentiate POAG cases from controls	Palmitoylcarnitine, hydroxyergocalciferol, sphingolipids, vitamin D-related metabolites, and terpenes were identified. Pentadecanone Heptadecanone, Heptadecanediol, Ergostanol Heptadecylbenzenediol, HODE-methyl ester; monoglyceride, Ergosterol were upregulated while few others downregulated (Multiple vitamin D analogs, sphingosine 1-phosphate, Complex glycosphingolipid). Alteration in steroid biosynthesis pathways was also indicated	PCA, PLS-DA, FDR, Two-way hierarchical cluster analysis (HCA)	Burgess et al. (2015)
Exfoliation glaucoma	Plasma	n = 205	n = 205	6 months		Untargeted	A total 379 metabolites were discovered; 33 metabolites were nominally significant	20 metabolites were nominally significant, including acetaminophen, methionine sulfoxide, and LPE, which were all positively associated while others were negatively correlated, cortisone has a strong inverse associated with XFG	Multivariable logistic regression	Kang et al. (2022)
Pseudo exfoliation glaucoma	Aqueous humor	n = 31 pseudo exfoliation glaucomatous patients	n = 41 (16 POAG & 25 non-glaucomatous controls)	cross-sectional	H NMR & LC-MS/MS	Not specified	A total of 298 metabolites in PEXA, POAG and non-glaucomatous controls 125 unique for PEXA, 63 unique POAG and 100 common metabolites	11 significant metabolites identified in PEXG (L-arginine, L-lysine, L-glutamine, L-tyrosine, 2,4-diacetamido-2,4,6-trideoxy-beta-l-altrose, N (6)-acetonyllysine, 1-aminocyclopropane-1-carboxylate, L-histidine, C6H9N4O3P, C6H13NO9, and 5 hydroxypentanoate). 2 metabolites (propylene glycol, creatine) were found to be significant using 1H NMR spectroscopy. L-arginine, L-lysine, L-tyrosine, L-glutamine, L-histidine showed varying levels when comparing PEX, POAG, and control. In H NMR, 2-hydroxybutyrate, 3-methyl-2-oxovalerate, propylene glycol, 3-hydroxy isovalerate, pyruvate, and choline had lower abundance in PEX compared to POAG	PCA, PLS-DA,SVM	Myer et al. (2020a)
Primary open-angle glaucoma (POAG)	Aqueous humor (AH)	n = 23 POAG patients	n = 35 controls	cross-sectional	HNMR and HPLC-MS	Not specified	278 in control 273 in POAG and 77 in common) metabolites identified using NMR 206 metabolites identified in LC-MS/MS	28 significant metabolites were discovered (NMR &LC-MS). Only 5 metabolites were found unique in POAG and 10 in the control. In POAG patients, significant increase in abundance of 4 amino acids (lysine, arginine, cysteine, and glycine), anthranilate, ascorbate, 4-hydroxybenzoate, myo-inositol, acetate, propylene glycol, 2-hydroxy-butyrate, creatine, and choline were observed. But phenylalanine, glutamine, 4-aminobutanoate, and isopropanol were decreased	PCA, PLS-DA	Myer et al. (2020b)
POAG	Plasma	n = 36 POAG patients	n = 27 age and sex matched controls with cataract	cross-sectional	LC-FIA-MS/MS	Targeted	151 metabolites identified	18 discriminant metabolites belonging to the carbohydrate, acyl-carnitine, phosphatidylcholine, amino acids, and polyamine families were discovered. 5 metabolites downregulated (spermidine, spermine, octadecadienyl-carnitine, octadecenoyl-carnitine and histamine) while 13 metabolites upregulated (tyrosine, methionine, the group of hexoses, phosphatidylcholine acyl-alkyl, methionine sulfoxide, propionyl-carnitine, three phosphatidylcholines, butyrylcarnitine, decenoyl-carnitine, dodecenoylcarnitine and arginine)	LASSO, MCUV, ROC, AUR, PCA, PLS-DA	Leruez et al. (2018)

#### 6.2.1 Pathway analysis of metabolites for glaucoma

**FIGURE 4:**
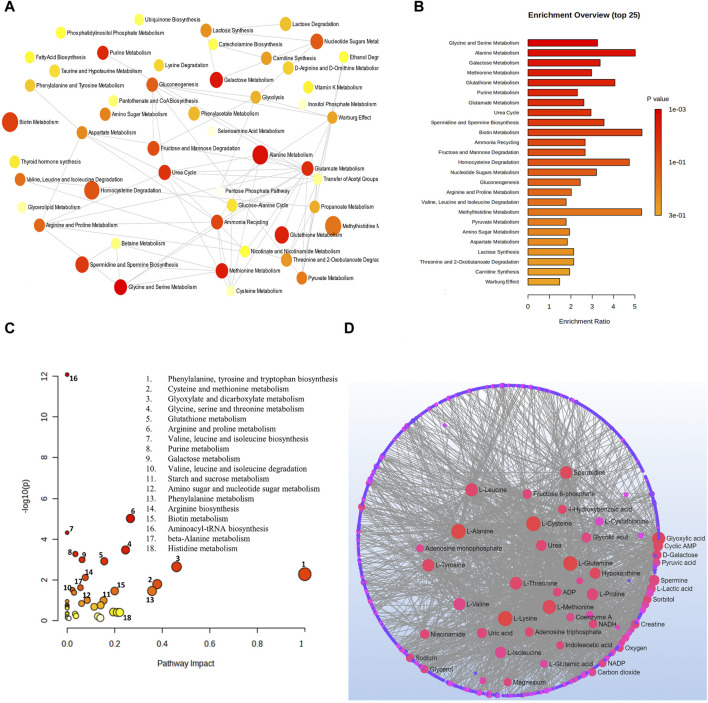
**(A)** Network view of metabolic pathway enrichment for glaucoma, **(B)** Summary plot of over-representation analysis (ORA) of metabolic pathways associated with glaucoma, **(C)** Overview of pathway impact analysis for glaucoma. The color of the circle indicates the significance level in the enrichment analysis, while the size of the circle reflects the pathway impact value from the topology analysis. **(D)** Metabolic-metabolic interaction network of glaucoma. The metabolite-metabolite interaction network allows for the exploration and visualization of interactions among functionally related metabolites.

Analysis combining metabolomic data from 10 glaucoma studies for metabolic pathway enrichment at a 0.05 significance level was conducted using MetaboAnalyst. This enrichment of metabolites was performed for samples taken from the vitreous, plasma, and serum that pointed to metabolic dysregulations in glaucoma patients. The pathway enrichment and pathway network topology analysis revealed that 14 metabolic pathways related to glaucoma, including glycine and serine metabolism, alanine metabolism, galactose metabolism, methionine metabolism, glutathione metabolism, purine metabolism, glutamate metabolism, urea cycle, spermidine and spermine biosynthesis, biotin metabolism, ammonia recycling, fructose and mannose deregulation, homocysteine deregulation and nucleotide sugar metabolism were significant (Figures 4A,B). Metabolic pathway impact and metabolic-metabolic interactions were figured out and displayed in Figures 4C,D, respectively.

### 6.3 Diabetic retinopathy

Diabetic retinopathy (DR) is a prevalent complication of diabetes mellitus and a significant contributor to visual impairment among middle-aged and elderly individuals (Wong et al., 2016). This condition is categorized into non-proliferative (NPDR) and proliferative (PDR) forms. NPDR is distinguished by the presence of microaneurysms, retinal hemorrhages, intraretinal microvascular abnormalities, and alterations in venous caliber, while PDR is characterized by the development of pathological pre-retinal neovascularization. Without proper management, this condition can lead to vision impairments and even blindness (Wong et al., 2018). Metabolomics is an essential tool in unraveling the pathophysiology of DR and discovering biomarkers to detect it early and provide specialized treatment. Specific metabolic changes have been identified through the examination of the metabolic profile of DR patients compared to those without the disease (He et al., 2023).

In 2016, Xuan and colleagues discovered metabolic alterations associated with DR, such as changes in amino acid metabolism, lipid metabolism, and energy metabolism (Xuan et al., 2020). The vitreous fluid of patients with DR showed multiple changes in these metabolic pathways and disruptions in the regulation of amino acid metabolism. In particular, there were heightened proline, phenylalanine, and tyrosine concentrations in the vitreous humor of patients with advanced RD relative to the comparison group (Tomita et al., 2021).

Significant changes extend beyond amino acids within DR, as lipid metabolism is also affected. Lipid accumulation can arise when lipid metabolism is unduly altered. Research has noted various lipid species have changed within the vitreous humor of patients with DR which may contribute to the presence of inflammation, stress due to oxidation, and vascular dysfunction which are observed in DR (López-Contreras et al., 2020). The exploration of the role of energy metabolism using metabolomics showed changes in metabolites relating to energy metabolism, including lactate, pyruvate, and acetyl-CoA, specifically in vitreous fluid from patients with DR. These shifts imply a direction towards anaerobic metabolism and mitochondrial dysfunction in retinal cells (Hou et al., 2021).

The biomarkers extracted from serum samples exhibited significant indications of perturbed metabolic pathways, specifically those related to glycine, serine, threonine, urea cycle, taurine, hypotaurine, cysteine, and methionine (Guo et al., 2022; Yousri et al., 2022; Shen et al., 2023). In case-control and longitudinal studies utilizing plasma samples, disrupted metabolisms of amino acids, leukotrienes, niacin, pyrimidine, purine, arginine, citrulline, glutamic semialdehyde, dehydroxycarnitine, 2,4-dihydroxybutyric acid, ribonic acid, ribitol, and 3,4-Dihydroxybenzoic acid have been identified (Sumarriva et al., 2019; Curovic et al., 2020). Moreover, L-Citrulline, indoleacetic acid, 1-methylhistidine, 1-phosphatidylcholines, hexanoylcarnitine, chenodeoxycholic acid and eicosapentaenoic acid emerged as the most discerning metabolic biomarkers for distinguishing the severity of DR in T2DM (Wang et al., 2022a). In untargeted metabolomics of T1DM patients, 3,4-DHBA was established as an independent risk marker for DR stage progression (Curovic et al., 2020).

Vitreous-based studies revealed a broad spectrum of upregulated metabolites including allantoin, lactate, proline, dimethylglycine, α-ketoglutarate and pyruvate (Tomita et al., 2021), while ascorbate, 5-oxoproline, and fumarate exhibited decreased levels alongside a downregulation of glycolysis and activation of the pentose phosphate pathway (Haines et al., 2018). Additionally, T1DM patients demonstrated a high concentration of lactate followed by glucose, alanine, valine, glutamine, acetate, leucine, isoleucine, and succinate (Barba et al., 2010).

Metabolic pathways involving carnitine, tryptophan, primary bile acid biosynthesis, pantothenate and CoA biosynthesis, glutathione, glycine, serine, threonine, cysteine, pentose phosphate, methionine, and aminoacyl-tRNA biosynthesis were significantly linked with DR (Chen et al., 2016; Sumarriva et al., 2019; Wang et al., 2022).

Thus, the discovery of these metabolic changes in DR carries important implications for the diagnosis and treatment of this condition. Metabolomic analysis offers a promising, non-invasive approach in detecting and classifying the risk associated with this disease at an early stage. By pinpointing distinctive metabolic patterns, healthcare professionals can enhance their ability to manage the DR effectively and apply precise interventions tailored to each patient’s needs. Additionally, metabolomics holds the potential to identify innovative targets for therapeutic intervention, paving the way for novel treatment strategies (Gonzalez-Covarrubias et al., 2022). The summary of significant metabolites and metabolic pathways, study characteristics, and analytical techniques of DR studies of human subjects is described in Table 3.

**TABLE 3:** Summary of metabolomics studies on DR, investigating metabolites in human biofluids, methodologies and analytical techniques.

Diseases/Condition	Sample/sample sources	Case(treatment) group	Control group	Type/study duration	Analytical technique	Metabolomics technique	Number of identified metabolite	Key findings	Evaluation standards	References
PDR	Vitreous	n = 43 PDR patients (age 27–80 years)	n = 21 nondiabetic control	2 Years	Waters ACQUITY UHP LC-MS/MS		158 metabolites were altered in PDR patient’s	Allantoin, lactate, proline, dimethylglycine, α-ketoglutarate, and pyruvate were increased in the VH of PDR patients compared with ERM patients Creatine levels were decreased in PDR patients Significant enrichment was observed in glycine, serine, arginine, and proline amino acid metabolism	PCA, FDR, mean SEM, unpaired *t*-test	Tomita et al. (2021)
DR	Plasma	n = 83 Patients with DR	n = 90 diabetic controls without DR	cross-sectional	LC-ESI Tandem-MS	Untargeted	151 features were identified, and 126 metabolites differed significantly	Alterations in the metabolism of amino acids, leukotrienes, niacin, pyrimidine, and purine. Arginine, citrulline, glutamic semialdehyde, and dehydroxycarnitine were observed b-oxidation of saturated fatty acids, fatty acid metabolism, and vitamin D3 metabolism pathways were altered and carnitine was a major contributor to the pathway differences	MWAS, PLS-DA, *t*-test	Sumarriva et al. (2019)
DR	serum and plasma	n = 48 individuals with T1D	no control	2 years	GC-TOF-MS	Untargeted	A total of 75 metabolite species were identified	4 metabolites were positively correlated to the baseline DR stage (2,4-dihydroxybutyric acid (DHBA), ribonic acid, ribitol and 3,4-DHBA. The four metabolite levels were significantly increased by higher DR stage 3,4-DHBA as an independent risk marker for progression in DR stage	ANCOVA, Multivariate linear regression, Partial regression	Curovic et al. (2020)
DR	Serum	N = 858 T2D patients n_1_ = 574 Qatari adults n_2_ = 282 from the Qatar BioBank	N = 2758 adults n_1_ = 422 Qatari adults n_2_ = 2336 from the Qatar BioBank	cross-sectional	not mentioned	Untargeted	373 metabolites were identified, 161 of which were novel metabolites	229 metabolites significantly associated with T2D (84 lipids, 42 amino acids, 11 carbohydrates, 11 peptides, 10 nucleotides, 6 xenobiotics, 4 cofactors and vitamins, 1 energy metabolite, and 60 unknown metabolites). Novel metabolites like thioproline, 3-methylglutaconate, dipeptides leucylalanine, phenylalanylglycine and threonylphenylalanine were identified. Metabolic pathway of N-methylproline and N-acetylarginine from the urea cycle; theobromine from xanthine metabolism; N-acetyltaurine, N-acetylmethionine, and S-methylcysteine from the methionine–taurine–S-adenosylmethionine (SAM) were discovered	PCA, Partial correlation	Yousri et al. (2022)
DR	Plasma	n = 40 diabetes patients with DR 40	n = 40 diabetes patients without DR	2 years	GC-TOF-MS Agilent 6890N	Untargeted	263 peaks were identified	11 metabolites were found to be correlated with DR. The metabolite markers of 2-deoxyribonic acid; 3,4-dihydroxybutyric acid; erythritol; gluconic acid and ribose were validated. 2-Deoxyribonic acid and 3,4-dihydroxybutyric acid are novel metabolite markers. Pentose phosphate pathway was identified as a key metabolic dysregulation associated with DR.	PCA, Mann-Whitney U tests, FDR, AUR, ROC and OR	Chen et al. (2016)
PDR	Plasma and Vitreous	n = 139(PDR patients 88 plasma and 51 vitreous samples)	n = 74(non-diabetic, 51 plasma, 23vitreous samples)	2 years	UPLC-MS/MS Ultimate 3000 UHPLC HSS T3 column	Untargeted	345 metabolites were detected in the plasma of PDR nondiabetic patients but 270 metabolites were identified from vitreous samples	From plasma, 15 differential metabolites and 7 metabolic pathways (tryptophan metabolism; primary bile acid biosynthesis; pantothenate, and CoA biosynthesis; glutathione metabolism; glycine, serine, and threonine metabolism; cysteine and methionine metabolism; and aminoacyl-tRNA biosynthesis) were identified. In vitreous 76 features that differentiated patients with PDR, metabolic pathways of pantothenate and CoA biosynthesis; pyrimidine metabolism; valine, leucine, and isoleucine biosynthesis; and phenylalanine metabolism were identified. 5 overlapping metabolites	PCA, OPLS-DA Mann–Whitney *U* test, *t*-test, FDR	Wang et al. (2022b)
DR	Serum	n = 195 T2DM- Both with DR and without DR	n = 755 healthy controls	10 months	UPLC-MS/MS	Targeted	613 metabolites were identified (318 in positive ion modes & 295 in negative ion modes)	89 metabolites were determined as DEMs (34 were under-regulated and the other 55 were increased) in DR. The biosynthesis of unsaturated fatty acids, thiamine metabolism, and glycine, serine, and threonine metabolism were detected as the significantly enriched pathways, and the hub metabolite (linoleate) of linoleic acid metabolism was manifestly decreased	PLS-DA, FDR, *t*-test	Guo et al. (2022)
DR with hard exudates	Serum	n = 116(both NPDR and PDR) patients	n = 51(validation group)	3.75 years	LC-MS	Untargeted	19 metabolites and 13 pathways associated with hard exudates of DR were identified	Methionine, Gamma Hydroxybutyric Acid, Salicylic acid, Citric acid, Tartaric acid, Oxalic acid, S-Adenosylhomocysteine, Ethylmalonic acid Ursodeoxycholic acid, Diethanolamine, Deoxycholic acid, Deoxycholic acid, Malonic acid Guanosine 3′,5′-Monophosphate, Malic acid, Taurine, L-3-O-Methyl-DOPA L-Cysteine and Hydroxypyruvic acid were identified. Taurine and hypotaurine metabolism, cysteine, and methionine metabolism were closely related to hard exudates	PCA, PLS-DA, *t*-test, ROC, AUC	Shen et al. (2023)
PDR	Vitreous	n = 22 (T1DM with PDR)	n = 22 with macular hole	cross-sectional	H-NMR	Not specified	14 significant metabolites were identified	high presence of lactate followed by metabolites of glucose, alanine, valine, glutamine, acetate, leucine, isoleucin, and succinate were identified	PCA, PLS-DA	Barba et al. (2010)
DR	Plasma	n = 83 T2DM patients	n = 27 matched controls	11 months	UHPLC-MS/MS (FIA-MS/MS)	Targeted	201 biomarkers significantly distinguished	57 biomarkers were higher in T2DM group, while the other 8 were lower than the control L-Citrulline, indoleacetic acid, 1-methylhistidine (1- phosphatidylcholines, hexanoylcarnitine, chenodeoxycholic acid, and eicosapentaenoic acid were the most distinctive metabolites biomarkers to distinguish the severity of DR	ANOVA, OPLS-DA FC, ROC, AUC	Wang et al. (2022a)
DR with RRD	Vitreous	n = 34(25 RRD, 9 PDR)	n = 8 controls	cross-sectional	UHPLC−MS	Untargeted	150+ metabolites were identified	Ascorbate, 5-oxoproline, and fumarate in the diabetic vitreous were decreased along with increased levels of proline, citrulline, and aspartate in the diabetic group. Downregulation of glycolysis accompanied by activation of the pentose phosphate pathway	ANOVA, PLSDA, ROC	Haines et al. (2018)
DR	Aqueous humor (AH)	n = 27(14 T2DM and cataract, 13 with DR and cataract)	n = 7controls with senile cataract	cross-sectional	H-NMR	Not specified	25 principal metabolites in the AH samples were identified	Lactate, succinate, 2-hydroxybutyrate, asparagine, dimethylamine, histidine, threonine, and glutamine were the most altered metabolites that potentially play roles in the development and progression of DR. The highly activated alanine, aspartate, and glutamate metabolic pathway were selected using pathway analysis in DR.	PCA, OPLS-DA, ROC	Jin et al. (2019)
DR with Macular Edema (ME)	Aqueous humor (AH)	n = 60 patients	n = 20 age matched	cross-sectional	HPLC-tandem MS	Untargeted	310 metabolites were identified	5 metabolites were positively correlated to the baseline DR stage (2,4-DHBA, ribonic acid, ribitol, and 3,4-DHBA. The four metabolite levels were significantly increased by higher DR stage	Mann–Whitney *U* test	Jiang et al. (2023)

#### 6.3.1 Pathway analysis for metabolites related to DR

**FIGURE 5:**
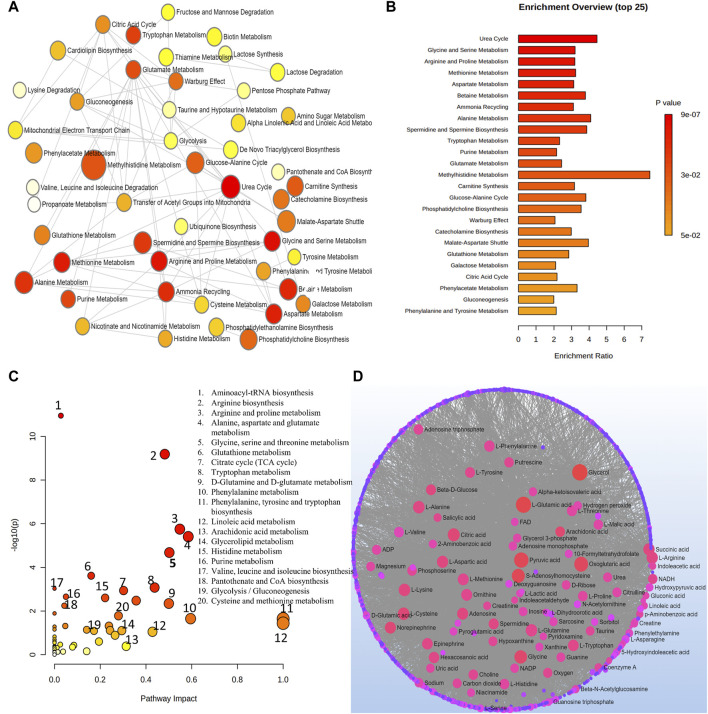
**(A)** Network view of metabolic pathway enrichment for DR. **(B)** Summary plot of over-representation analysis (ORA) of metabolic pathways associated with DR. **(C)** Overview of pathway impact analysis for DR. The color of the circle indicates the significance level in the enrichment analysis, while the size of the circle reflects the pathway impact value from the topology analysis. **(D)** Metabolic-metabolic interaction network of DR. The metabolite-metabolite interaction network allows for the exploration and visualization of interactions among functionally related metabolites.

At a level of significance of 0.05 (*p*-value), the analysis of important metabolites from 13 DR studies was done to look for metabolic pathway enrichment. Samples from aqueous and vitreous humor, plasma, and serum that indicated metabolic dysregulations in patients with DR were subjected to notable enrichment of metabolites. The results of the pathway enrichment and pathway network topology analysis indicated that there were 13 significant metabolic pathways associated with DR. These pathways include the metabolisms of urea cycle, glycine and serine, arginine and proline, methionine, aspartate, betaine, ammonia recycling, alanine, spermidine and spermine biosynthesis, tryptophan, purine, glutamate and methylhistidine (Figures 5A,B). Figures 5C,D illustrate the impact of metabolic pathways and metabolic-metabolic interactions, respectively. It has been shown that the development and progression of DR are associated with the deregulation of certain metabolic pathways. This implies that treatment strategies for the management of DR may benefit from targeting these pathways.

### 6.4 Myopia

Myopia is the most prevalent refractive abnormality, affecting approximately 4.76 billion people who are expected to suffer from myopia by the year 2050, which represents half of the world’s population. The trend of myopia is predicted to increase from 27% of the world’s population in 2010 to 52% by 2050. In 2000, the prevalence of high myopia was 2.7%, and it is projected to 9.8% by the year 2050 (Holden et al., 2016). Numerous variables, including ethnic/genetic predisposition, crowded environments, early schooling, near-distance activities, and less time spent outdoors, are believed to increase the risk of myopia development (Rudnicka et al., 2016). The prevalence of myopia varies greatly across communities, age groups, and even persons with different educational levels, and it has been continuously rising over the last several decades (Holden et al., 2016). Because the underlying molecular, physiological, and anatomical changes are irreversible, identifying the genetic basis and environmental factors that contribute to the development of myopia (She et al., 2021) has meaningful implications for reducing the growing public health burden associated with myopia.

In myopic eyes, the molecules generated by cellular metabolism and their concentrations provide insight into the underlying biological processes at play (Yu et al., 2020). It is a fact that innovative research methodologies may give a better insight into the etiology of myopia, which could ultimately encourage developments in its prevention and treatment. Progress in analytical techniques and constant enhancement of computing skills allowed for the development of metabolomics approaches. The use of metabolomics offers a holistic approach for comprehending the molecular alterations associated with myopia (Lauwen et al., 2017). Anticipating the early identification of individuals at high risk for myopia and their associated pathological retinal complications could lead to a more personalized approach to myopia treatment. Studies have demonstrated differences in the levels of specific amino acids, lipids, and carbohydrates between myopic and non-myopic eyes. One potential application of metabolomics in myopia research is identifying biomarkers for the condition. These biomarkers are measurable indicators of a disease that can be used to diagnose, monitor, or predict myopia progression (Lian et al., 2022).

Aqueous-based investigations of myopia with the help of UHPLC-QTOF-MS depicted a range of changes among metabolites (aminocyclohexanecarboxylic acid, amino octanoic acid, L-arginine citrulline, amino undecanoic acid, dodecanedioic acid, and butyryl-L-carnitine pantothenic acid) during the course of the disease. These changes in amino acids contributed to the development and progression of myopia by affecting the structure and function of the retina. The key metabolite dysregulates the normal physiological and impulse signaling of the eye (Barbas-Bernardos et al., 2016).

MS-based analysis of VH samples from myopic patients elucidates differentially expressed metabolites and their corresponding abundance levels. Metabolic groups of amino acids, lipids, purines, organic acids, organooxygen, organonitrogen, and indoles were expressed differently in the myopic group (Tang et al., 2023). The concentrations of glutamine, hypoxanthine, decanoylcarnitine, Dl-glutamic acid, citrulline, and pyroglutamic acid were significantly higher while prostaglandin G2, L-threonic acid, and citramalate showed lower concentrations (Ji et al., 2017).

On the other hand, samples from serum confirmed the dysregulation of many metabolites and altered metabolic pathways. The GC-TOF-MS analysis of serum revealed the downregulation of alanine, mannose, itaconic acid, aconitic acid, O-acetylserine, phthalic acid, abietic acid, and salicin. Conversely, metabolites such as citric acid, aminomalonic acid, palmitoleic acid, conduritol-b-epoxide, shikimic acid, 4-hydroxyphenylacetic acid, hesperitin, anandamide, oxalacetic acid, pimelic acid, 2-ketoadipate and N-ethylmaleamic acid were upregulated (Ke et al., 2021). The summary of significant metabolites and metabolic pathways, study characteristics, and analytical techniques of myopia studies of human subjects is described in Table 4.

**TABLE 4:** Summary of metabolomics studies of myopia, investigating metabolites in human biofluids, methodologies and analytical techniques.

Diseases/Condition	Sample/sample sources	Case(treatment) group	Control group	Type/study duration	Analytical technique	Metabolomics Techniques	Number of identified metabolites	Key findings	Evaluation standards	References
Pathological myopia (PM)	Vitreous	n = 39 PM patients with RRD (23) or macular hole (MH) (16)	n = 23 controls /moderate myopia	Cross sectional	UPLC‒MS	Untargeted	23 metabolites altered (both positive and negative modes)	Amino acids, lipids, purines, organic acids, organooxygen, organonitrogen, and indoles were significantly altered in PM group. L- carnitine, Propionylcarnitine, Theophylline, Theobromin, Uric acid, and estradiol were upregulated but N-acetylhistidine, 16 Methylheptadecanoic acid and indoxyl sulphate were downregulated in PM. Tryptophan metabolism and uric acid were closely correlated	PCA, OPLS-DA, FDR, ROC, AUR	Tang et al. (2023)
High myopia (HM)	Serum	n = 30 high myopia cases	n = 30 controls (non-myopic)	Cross sectional	LC-QTOF/MS	Untargeted	9 Unique metabolites were identified	Seryltryptophan, 5-methyltetrahydrofolic acid, phosphatidylethanolamine (PE), lysophosphatidylethanolamine (LysoPE), 25-hydroxyvitamin D2-25 glucuronide, γ-glutamyltyrosine, 12-oxo-20-trihydroxy-leukotriene B4, and 5-hydroxytryptamine were identified. 7 metabolites showed significantly higher concentrations, and 2 metabolites were lower in the myopia subjects	PCA, PLS-DA	Dai et al. (2019)
Myopia	Aqueous humor	n = 37 (12 HM, 24 LM, and 1 with no myopia for method development)	No control	Cross-sectional	UHLC-QTOF-MS Capillary electrophoresis–MS (CE–MS)	Untargeted	44 metabolites were identified in human AH	Aminocyclohexanecarboxylic acid, aminooctanoic acid, L-arginine citrulline, aminoundecanoic acid, dodecanedioic acid, butyryl-L-carnitine pantothenic acid, trihydroxyphenyl-gamma-valerolactone, didehydro-retinoic acid, sphinganine, L-cysteinylglycine disulfide, histidinyl-phenylalanine, dihydropteroic acid, dimethylnonanoyl carnitine, sulfatide and trihexosylceramide were significant in ah of myopia	PCA, OPLS-DA	Barbas-Bernardos et al. (2016)
High myopia (HM)	Aqueous humor	n = 20 with high myopia	n = 20 controls	Cross-sectional	GC- TOF MS	Untargeted	242 metabolites were identified in AH	34 amino acids, 44 carbohydrates, 9 lipids, 7 nucleotides, and other 148 metabolites (e.g., glutamine 1, N-alpha-Acetyl-L-ornithine 3, Nicotinoylglycine 2, o-Hydroxyhippuric acid 2 oxalacetic acid, oxalic acid ribose, cis-gondoic acid) were identified. 29 metabolites changed based on significant correlation (27 metabolites upregulated and 2 decreased)	PLS-DA	Lian et al. (2022)
Myopia	Serum	n = 108 myopic cases	n = 103 controls	Cross-sectional	Vanquish UHPLC-MS	untargeted	275 metabolite features were derived and 33 pathways mapped	16 metabolites from positive and 173 from negative ionization mode were significant (both groups). 66 metabolites were found to be significantly different between cases and controls. Significant metabolic pathways of steroid biosynthesis b, lysine degradation, arginine, and proline, glycerolipid, glycerophospholipid, arachidonic acid, linoleic acid, alpha-linolenic acid, and sphingolipid metabolism were identified	YU test, FC, Screening rank	Du et al. (2020)
high myopia	Serum	n = 40 adults with high myopia	n = 40 adults with low myopia	Cross-sectional	GC-TOF-MS	Untargeted	20 metabolites were identified as potential biomarkers of high myopia	Alanine, mannose, itaconic acid, aconitic acid, O-acetylserine 1, phthalic acid, abietic acid, and salicin were decreased in HM group but citric acid, aminomalonic acid, palmitoleic acid, conduritol b epoxide, shikimic acid, 4-hydroxyphenylacetic acid, hesperitin, anandamide, oxalacetic acid, oxalacetic acid, pimelic acid, 2-ketoadipate and N-ethylmaleamic acid were increased. 7 pathways were significantly enriched (citrate cycle; selenoamino acid, alanine, aspartate, and glutamate metabolism; glycolysis, gluconeogenesis; glyoxylate anddicarboxylate metabolism; cysteine and methionine and biotin metabolism)	PCA, OPLS-DA, AUC	Ke et al. (2021)
pathological myopia (PM)	Aqueous humor and vitreous humor	n = 30 p.m. patients	n = 30 non- myopic control	Cross-sectional	Vanquish UHPLC-MS/MS	Both untargeted and Targeted	508 metabolites in the AH and 601 in the VH were identified	218 metabolites were significant. SQH, inosine, uridine, hypoxanthine, 3-nitro-L-tyrosine, phosphopyruvic acid, DL-otyrosine, and APK were significantly higher, while pseudouridine and L-threo-3-phenylserine showed lower concentrations in AH. D-(−)-Glutamine, hypoxanthine, decanoylcarnitine, Dl-Glutamic acid, L-(+)-Citrulline and L-Pyroglutamic acid were significantly higher while prostaglandin G2, L-threonic acid, citramalate, and KPH) showed lower concentrations in VA of PM group. Bile secretion, insulin secretion, thyroid hormone synthesis &cGMP-PKG signaling pathway were identified	PLS-DA, FC	Ji et al. (2017)
Myopic Retinopathy	Serum	n = 185 myopic retinopathy	n = 331 controls	8 Months	GC–TOF–MS	Untargeted	390 named metabolites were identified	2-HBA, 2-hydroxy-2-MBA, 3-HBA, ribitol, hypoxanthine, phosphoethanolamine, stearic acid, homoserine, linoleic acid, glycolic acid, maleimide, glycerol, and N-carbamoylaspartate) decreased while proline, resorcinol, citric acid, isolinoleic acid, docosenoic acid, valine, oxamic acid, isothreonic acid, citrulline, pinitol, histidine, monomyristin, and 1-monoheptadecanoyl glyceride) were significantly increased. 62 metabolic pathways, mainly involving amino acid and carbon dioxide metabolism-related pathways detected	ROC, AUC, SVM	Hou et al. (2023b)

#### 6.4.1 Pathway analysis for metabolites related to myopia

**FIGURE 6:**
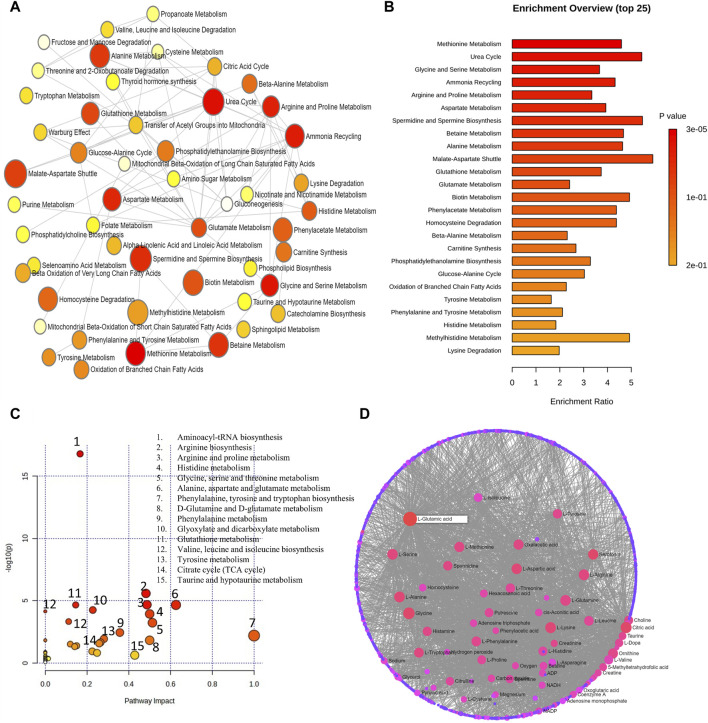
**(A)** Network view of metabolic pathway enrichment for myopia, **(B)** Summary plot of over-representation analysis (ORA) of metabolites associated with myopia. **(C)** Overview of pathway impact analysis for myopia. The color of the circle indicates the significance level in the enrichment analysis, while the size of the circle reflects the pathway impact value from the topology analysis. **(D)** Metabolic-metabolic interaction network of myopia. The metabolite-metabolite interaction network allows for the exploration and visualization of interactions among functionally related metabolites.

The metabolic pathway enrichment analysis of 8 human metabolomics studies pertaining to high myopia on differential metabolites from aqueous, vitreous, and serum samples revealed 12 significant metabolic pathways. These metabolic pathways consist of methionine, urea cycle, glycine and serine, ammonia recycling, arginine and proline, aspartate, spermidine and spermine biosynthesis, betaine, alanine, malate aspartate shuttle, glutathione, and glutamate metabolisms (Figures 6A,B). Figures 6C,D demonstrate the impact of metabolic pathways and metabolic-metabolic interactions in myopia, respectively.

### 6.5 Differential metabolites/potential biomarkers across common ocular disorders

Elucidating common metabolic biomarkers for multifactorial ocular disorders is not a trivial endeavor that may be subjected to misleading inferences. Keeping all the diverse variations in sample sources, disease stages, and condition heterogeneity, certain metabolites have been identified as common to various ocular disorders. Glycine, lysine, cysteine, alanine, glycerol, and methionine were among the differential metabolites found in common among the major ocular conditions. The degree of significance and regulation of these metabolites in each disease condition varies considerably depending on the underlying causes, souces of the sample, extraction technique and the stage of the disease.

## 7 Discussion

This review has highlighted significant metabolic changes in common multifactorial ocular disorders, including glaucoma, DR, AMD, and myopia. Identifying these metabolic changes and their common pathways plays a crucial role in the progression and development of these ocular disorders.

The prominent metabolites altered in the pathogenesis process of AMD range from amino acids (acetylasparagine, a component of alanine and aspartate metabolism) to lipids (cholesterols, glycerides, and phospholipids), nucleotide polymorphisms, and vitamin D-related metabolites. Studies employing ultra-high-performance liquid chromatography-tandem MS on AMD patients and controls discovered substantial variations in plasma metabolites. On the other hand, lipid profiles, followed by amino acid and nucleotide profiles, showed the most variance (Lains et al., 2018; Lains et al., 2019). Another study discovered substantial alterations in the metabolism of amino acids in AMD patients, including alterations in N-acetyl-L-alanine, L-tyrosine, L-phenylalanine, L-methionine, and L-arginine (Luo et al., 2017). In targeted metabolomic analysis, patients with increased plasma omega-3 LC-PUFA levels had a lower chance of developing neovascular AMD (Wang et al., 2016).

In this review, the authors performed pathway analysis using the list of compound names extracted from eligible studies. In the pathway analysis considering their *p*-values, the top 15 impact metabolic pathways belong to lipid metabolism, primary bile acid biosynthesis, purine metabolism, arginine and proline metabolism, steroid biosynthesis, d-glutamine and d-glutamate metabolism, taurine and hypotaurine metabolism, nicotinate and nicotinamide metabolism, glycerolipid metabolism, steroid hormone biosynthesis, tryptophan metabolism, galactose metabolism, glycerophospholipid metabolism and glutathione metabolism. The result of this pathway enrichment is consistent with other studies that have discovered comparable metabolic pathways linked to arginine and proline metabolism, mitochondrial synthesis, purine metabolism, thiamine metabolism, methionine metabolism, fatty acid metabolism, steroidogenesis, alanine metabolism, butyrate metabolism, and vitamin B6 metabolism (Hou et al., 2020). This implies that figuring out the metabolic pathways and the regulation-specific mechanisms involved in the pathophysiological process of AMD and other multifactorial ocular disorders are important.

Another pathway analysis indicated substantial alterations in glycerophospholipid, purine, taurine, and hypotaurine metabolism, which may be linked to the activation of oxidative stress in AMD. Purine, taurine, and hypotaurine metabolic dysregulation may be associated with disrupted antioxidant defense, neuroprotection, and cellular energy metabolism deregulation (Lains et al., 2018; Lains et al., 2019). Changes in glutamine and glutamate levels between people with AMD and controls, as well as between different AMD severity stages, can be linked to problems with the neurotransmitter supply that is crucial for the retina and visual pathway (Lains et al., 2018).

Studying the metabolite profiles of aqueous humor and serum in glaucomatous patients can aid in identifying important indicators of physiological and pathological states, as well as elucidating the mechanisms of disease onset and progression. Metabolic markers play a crucial role in early detection and can differentiate disease stages. Quantitative analysis of amino acids (e.g., homocysteine), vitamin B12, and folic acid in the plasma of PEXG, POAG, and control subjects revealed higher plasma homocysteine levels in PEXG patients compared to both POAG and controls (Tranchina et al., 2011).

As revealed in this review, the top enriched metabolic pathways linked to glaucoma were metabolisms of glycine and serine, alanine, galactose, methionine, glutathione, purines, and glutamate. the urea cycle, spermidine and spermine biosynthesis, biotin, ammonia recycling, fructose, and mannose deregulation, homocysteine deregulation, and nucleotide sugar metabolism. The majority of these pathways were traced in a previous meta-analysis study of open-angle glaucoma from aqueous, plasma, and serum samples. The top spotted metabolic pathways were glycine, serine, and threonine metabolism, alanine, aspartate, and glutamate metabolism, taurine and hypotaurine metabolism, sphingolipid metabolism, arginine and proline metabolism, glutathione metabolism, glyoxylate and dicarboxylate metabolism, aminoacyl-tRNA biosynthesis and biotin metabolism, and beta-alanine metabolism (Wang et al., 2021).

Regarding DR, numerous significant metabolites of lipids, amino acids, carbohydrates, peptides, nucleotides, xenobiotics, cofactors, and vitamins were identified from the reviewed studies. Across different studies, some of the common metabolites which showed significant changes in DR case include: allantoin, lactate, proline, dimethylglycine, α-ketoglutarate, pyruvate, 2,4-dihydroxybutyric acid, ribonic acid, ribitol, erythritol; gluconic acid, methionine, gamma hydroxybutyric acid, salicylic acid, citric acid, tartaric acid, oxalic acid, S-adenosylhomocysteine, ethylmalonic acid, ursodeoxycholic acid, diethanolamine, deoxycholic acid, deoxycholic acid, malonic acid, guanosine 3′,5′-monophosphate, malic acid, taurine, L-3-O-Methyl-DOPA L-cysteine, hydroxypyruvic acid, 3,4-DHBA, thioproline, 3-methylglutaconate, dipeptides leucylalanine, phenylalanylglycine and threonylphenylalanine. Studies using plasma samples from DM patients with mild non-proliferative to proliferative DR were subjected to a metabolomics investigation employing the GC-MS technology, which identified significantly changed metabolites. There was a drop in 1,5-anhydroglucitol, whereas 1,5-gluconolactone, 2-deoxyribonic acid, 3,4-dihydroxybutyric acid, erythritol, gluconic acid, lactose/cellobiose, maltose/trehalose, mannose, ribose, and urea levels increased. A possible disease biomarker, cytidine, has also been identified (Chen et al., 2016; Wang et al., 2022; Wang et al., 2022c).

Methionine, allantoin, decanoylcarnitine, arginine, proline, citrulline, ornithine, and octanoylcarnitine dysregulation were found in individuals with proliferative DR after a global and targeted LC-MS metabolomic analysis of vitreous samples from patients with type 2 diabetes (Paris et al., 2016). The metabolites that have undergone the biggest alteration may contribute to the DR’s onset and progression. Many metabolic pathways were impacted, presumably due to mitochondrial malfunction, oxidative stress, and endothelial damage in individuals with DR, according to metabolic pathway analysis. Arginase pathway disruption may lead to DR-related endothelial dysfunction, decreased nitric oxide availability, poor vasodilation, and increased production of oxygen and nitrogen reactive species (Jian et al., 2022).

Eight myopia human metabolomics studies were included in this review study. The studies utilized samples of aqueous, vitreous, plasma, and serum. Pathway analysis identified twelve statistically significant metabolic pathways that were enriched. The serum metabolism of myopia showed a high enrichment of the TCA cycle and glyoxylate and dicarboxylate metabolism abnormalities, indicating a potential role for glycometabolism disorders in the pathophysiology of myopia. Ocular axial length and refraction have been shown to be coupled with the expression of metabolic genes linked to mitochondrial metabolism pathways, indicating an aberrant energy metabolism during the development of myopia (Riddell et al., 2016). Given that both AMD and DR have the same aberrant energy metabolism feature as high myopia, metabolic regulatory mechanisms regulating the development of myopia, DR and AMD seem to be related (Mullins et al., 2005; Hou et al., 2021). Other metabolites such as arginine, citrulline, and sphinganine were significantly altered in various myopic groups. (Barbas-Bernardos et al., 2016; Chen et al., 2018).

The metabolic pathways that demonstrated the highest degree of enrichment in the pathway analysis of myopia were methionine metabolism, urea cycle metabolism, glycine and serine metabolism, ammonia recycling, and arginine and proline metabolism. This finding was consistent with similar studies in which citrate cycle pathways, metabolism of aspartate, glutamate, and alanine; the metabolism of glyoxylate and dicarboxylate; and the biosynthesis of unsaturated fatty acids, exhibited similar degrees of enrichment (Hou et al., 2023a).

In the realm of ocular diseases, leveraging metabolomics data can be a powerful tool in unraveling shared metabolic pathways and identifying potential biomarkers for diagnosis and treatment. By employing statistical methods such as multivariate analysis or pathway enrichment analysis, researchers can account for confounding factors and distinguish disease-specific metabolic signatures from commonalities across different ocular conditions. Adjusting variables like age, gender, or medication usage can help refine the analysis and enhance the accuracy of identifying disease-specific metabolic alterations. This approach not only aids in understanding the underlying mechanisms of a particular ocular disease but also paves the way for personalized medicine strategies tailored to individual metabolic profiles.

The major limitation in metabolomics studies, truly unbiased analyses are challenging to achieve. The metabolite extraction protocol itself inherently introduces a degree of bias, as certain metabolites may be preferentially extracted or lost during the sample preparation process. As such, it is crucial to recognize that the metabolomics data presented represents a snapshot of the metabolome that is influenced by the specific extraction method employed.

## 8 Conclusion and future perspectives

In the analysis of metabolic interactions, glycine and adenosine monophosphate were consistently identified as the top metabolites in AMD, while methionine, lysine, alanine, glyoxylic acid, and cysteine were found to be frequently associated with glaucoma. In the context of myopia, glutamic acid, glycine, lysine, citric acid, alanine, and serotonin were observed to be highly networked metabolites. Notably, metabolites such as glycerol, glutamic acid, pyruvic acid, glycine, cysteine, and oxoglutaric acid were identified as significant in relation to DR. Among the pathways identified, arginine and proline metabolism, methionine metabolism, glycine and serine metabolism, urea cycle metabolism, and purine metabolism have been recurrently associated with these ocular conditions, highlighting potential commonalities in their metabolic underpinnings.

Recent developments in metabolomics methods have improved metabolic profiling considerably, offering important new insights into the metabolism and underlying causes of various ocular disorders. Biofluids and tissue samples are essential biological matrices for investigating ocular disorders, with metabolite associations between plasma and serum established for disorders including glaucoma, AMD, DR, and myopia. There is a great deal of promise in the developing discipline of metabolomics in eye research to identify biomarkers for disease detection.

Although metabolic research has advanced significantly in recent years, there are still issues that need to be resolved. The number of metabolites that have been annotated still remains small (not more than 30% of the detected molecular features can be identifiable and quantifiable) and there are inconsistent metabolite identifications (Muthubharathi et al., 2021). For metabolomics, the Human Metabolome Database (HMDB) database (Wishart et al., 2022), which is often used, still keeps extending the number of metabolites in which advancements contribute to widening the horizon.

Variations in sample preparation, selection and data analysis during an experiment may produce findings that are not comparable. It needs standardized methodologies for metabolomics analysis since many studies lack validation cohorts to identify possible biomarkers. On the other hand, to define the metabolic spectrum of disease controlling confounding variables including diet, gender, systemic and underlying conditions, large-scale prospective longitudinal studies are required. Metabolomics data may be affected by the co-occurring systemic disorders that patients often have. Researchers should make sure that clinical characteristics, such as blood pressure, blood cholesterol levels, and disease duration, are similar in order to guarantee a disease-specific metabolomics combination. The use of metabolomics in eye research is constrained by the inaccessibility and limited number of posterior segment tissue samples that are particular to the disease, which places increased demands on the sensitivity of metabolomics technology. Many ocular disorders have been treated by combining metabolomics and other omics, so multi-omics integration will bring a new layer of knowledge to our understanding of metabolic processes.
